# Exploring Microelement Fertilization and Visible–Near-Infrared Spectroscopy for Enhanced Productivity in *Capsicum annuum* and *Cyprinus carpio* Aquaponic Systems

**DOI:** 10.3390/plants13243566

**Published:** 2024-12-20

**Authors:** Ivaylo Sirakov, Stefka Stoyanova, Katya Velichkova, Desislava Slavcheva-Sirakova, Elitsa Valkova, Dimitar Yorgov, Petya Veleva, Stefka Atanassova

**Affiliations:** 1 Faculty of Agriculture, Trakia University, Students Campus, 6000 Stara Zagora, Bulgaria; st_stoyanova@mail.bg (S.S.); katya.velichkova@trakia-uni.bg (K.V.); elitsa.valkova@trakia-uni.bg (E.V.); dimitar.yorgov@trakia-uni.bg (D.Y.); petya.veleva@trakia-uni.bg (P.V.); stefka.atanasova@trakia-uni.bg (S.A.); 2 Faculty of Agronomy, Agriculture University, 4000 Plovdiv, Bulgaria; bioagro_pl@abv.bg

**Keywords:** aquaponic system, common carp (*Cyprinus carpio*), influence, pepper (*Capsicum annuum*), productivity, microelements

## Abstract

This study explores the effects of varying exposure times of microelement fertilization on hydrochemical parameters, plant growth, and nutrient content in an aquaponic system cultivating *Capsicum annuum* L. (pepper) with *Cyprinus carpio* (*Common carp* L.). It also investigates the potential of visible–near-infrared (VIS-NIR) spectroscopy to differentiate between treated plants based on their spectral characteristics. The findings aim to enhance the understanding of microelement dynamics in aquaponics and optimize the use of VIS-NIR spectroscopy for nutrient and stress detection in crops. The effects of microelement exposure on the growth and health of *Cyprinus carpio* (*Common carp* L.) in an aquaponic system are investigated, demonstrating a 100% survival rate and optimal growth performance. The findings suggest that microelement treatments, when applied within safe limits, can enhance system productivity without compromising fish health. Concerning hydrochemical parameters, conductivity remained stable, with values ranging from 271.66 to 297.66 μS/cm, while pH and dissolved oxygen levels were within optimal ranges for aquaponic systems. Ammonia nitrogen levels decreased significantly in treated variants, suggesting improved water quality, while nitrate and orthophosphate reductions indicated an enhanced plant nutrient uptake. The findings underscore the importance of managing water chemistry to maintain a balanced and productive aquaponic system. The increase in root length observed in treatments 2 and 6 suggests that certain microelement exposure times may enhance root development, with treatment 6 showing the longest roots (58.33 cm). Despite this, treatment 2 had a lower biomass (61.2 g), indicating that root growth did not necessarily translate into increased plant weight, possibly due to energy being directed towards root development over fruit production. In contrast, treatment 6 showed both the greatest root length and the highest weight (133.4 g), suggesting a positive correlation between root development and fruit biomass. Yield data revealed that treatment 4 produced the highest yield (0.144 g), suggesting an optimal exposure time before nutrient imbalances negatively impact growth. These results highlight the complexity of microelement exposure in aquaponic systems, emphasizing the importance of fine-tuning exposure times to balance root growth, biomass, and yield for optimal plant development. The spectral characteristics of the visible–near-infrared region of pepper plants treated with microelements revealed subtle differences, particularly in the green (534–555 nm) and red edge (680–750 nm) regions. SIMCA models successfully classified control and treated plants with a misclassification rate of only 1.6%, highlighting the effectiveness of the spectral data for plant differentiation. Key wavelengths for distinguishing plant classes were 468 nm, 537 nm, 687 nm, 728 nm, and 969 nm, which were closely related to plant pigment content and nutrient status. These findings suggest that spectral analysis can be a valuable tool for the non-destructive assessment of plant health and nutrient status.

## 1. Introduction

Aquaponic systems, an innovative fusion of aquaculture and hydroponics, have been lauded as a potential solution to various environmental and food security challenges [1]. This integrated multitrophic system combines the components of a recirculating fish culture system with hydroponics [2], with the water from the fish being used to stimulate plant growth. Aquaponic technology for food production for humans has evolved as a sustainable and environmentally friendly agricultural production system that maintains water quality and provides useful plant crops without pesticides [3]. For their normal growth and development, plant organisms require macro- and microelements (C, H, O, N, P, K, Ca, S, and Mg, as well as Fe, Cl, Mn, B, Zn, Cu, and Mo). Hydroponic solutions contain an optimized amount of these elements, with the exception of C, H, and O, which are available from air and water [4,5]. In aquaponics, plant nutrients are obtained from fish in the form of soluble organic compounds [4]. In order for plants to grow and develop normally, the availability of macro and micronutrients must be monitored periodically [4]. For example, iron is deficient in fish waste and may need to be added in a certain concentration [4,6,7]. Central to symbiosis relations in this innovative system is the transformation of fish waste into essential plant nutrients and the plants’ subsequent role in water purification, thereby benefiting the co-cultured aquatic species [8]. However, maintaining the fine-tuned balance of this intricate ecosystem requires a deeper understanding of several influencing factors such as water quality, nutrient dynamics, and environmental interplays.

Microelements, although required in minute quantities, have a profound influence on the overall health and productivity of both aquatic and plant components in an aquaponic system. Anderson et al. (2017), Delaide et al. (2017), and Guzel et al. (2018) have underscored the significance of these trace elements in supporting optimal plant growth [9,10,11]. Conversely, studies by Luo et al. (2021) and Cifuentes–Torres et al. (2021) have highlighted their critical role in fish health and development [12,13]. Microelements such as iron, zinc, and copper are crucial for a plant’s metabolic processes but are often present in insufficient amounts in fish waste, requiring careful supplementation to avoid deficiencies [14]. Excessive or imbalanced microelement levels can lead to toxicity, harming both plants and fish, emphasizing the need for precise nutrient management. Overall, optimizing microelement levels is key to maintaining system stability and promoting the health and productivity of both plants and fish in aquaponic systems.

Past research, including work by Aslanidon et al. (2023), has touched upon the potential reasons for microelement imbalances in aquaponic settings [15]. Yet, a comprehensive understanding of how varying the exposure time of a microelement impacts the system remains elusive.

Moreover, focusing on both pepper (*Capsicum annuum*), a popular choice among horticulturalists in aquaponic systems, and common carp (*Cyprinus carpio*), a fish species with significant global culinary and cultural value, provides a practical context. Rosta and Mohsenian (2021) and Saseendran et al. (2021) have previously explored the nutrient requirements and cultivation of pepper plants in aquaponics [16,17]. Concurrently, studies by Sirakov et al. (2021) and Asadi et al. (2021) have investigated the growth and physiological responses of common carp under aquaponic conditions [18,19].

The management of microelements in aquaponic systems is paramount to achieving optimal productivity. Microelements, also known as trace minerals, play vital roles in the metabolic processes of both fish and plants, ensuring healthy growth and development. Any imbalance or deficiency can hamper the symbiotic relationship between the aquatic and plant components, leading to reduced yields and system inefficiencies. Thus, the precise management of these essential nutrients is foundational to the success of any aquaponic endeavor. Based on this, we hypothesize that the duration of microelement treatment could significantly affect the productivity of an aquaponic system.

Monitoring the plant’s nutrient status when it is grown in aquaponics is very important to maintain the balance and sustainability of the system; this is because plant nutrients that are bioavailable from fish excreta are not enough for optimal plant growth. A classical method for estimating the status of nutrients is laboratory chemical analysis, which is destructive, includes chemical reagents, has lengthy processing times, and has a high cost. The development of reliable, rapid, and low-cost methods is very important.

Crop conditions could be estimated based on plants’ spectral characteristics in the visible and near-infrared regions. The nutritional status of plants and the symptoms of nutrient stress or diseases are manifested with changes in their color characteristics because of pigment content changes. This could affect the spectral pattern generated in the visible region from 400 to 700 nm. The structure of leaves and their water content define the spectra in the near-infrared region from 700 to 1100 nm. Hence, both regions have been recognized for plant nutrient status evaluation. Healthy plants showed low reflectance in the visible region and high reflectance in the near-infrared range.

Recently, spectroscopy and hyperspectral images in the visible and near-infrared regions were used to investigate the plants’ pigment content, nutrient status, or disease based on spectral reflectance [20,21,22,23,24]. Several authors reported pepper plant investigations. Hyperspectral imaging in the VIS-NIR region to examine the differences in the spatial distribution of chlorophyll values in leaves located at different positions on pepper plants has been reported [25]. The authors selected wavelengths, correlated to chlorophyll content [26]; investigated possibilities for the discrimination of pepper cultivars through field spectroscopy in the near-infrared region (700 to 1000 nm); and suggested that NIR spectroscopy measurements will be useful in integrated nutrient and disease management. The performance of several vegetable indices to estimate crop nitrogen status in sweet pepper has also been investigated [27]. Two reflectance sensors were used to measure canopy reflectance—one of them working at 660 and 780 nm, and the second at 550, 670, 760, and 730 nm. The best performance of the investigated indices was found in the early fruit growth and flowering stages. The highest coefficient of determination between vegetation indices and crop nitrogen in sweet pepper was obtained for green band-based indices [28] using a miniature fiberoptic spectrometer in the 200–1150 nm spectral range for the determination of water stress in greenhouse-grown bell pepper. The authors found that vegetative indices based on reflectance at 551, 553, 600, 670, 700, 800, 900, and 970 nm were the most useful indices for detecting water stress in bell pepper plants. A spectral assessment of two-spotted spider mite damage levels in the leaves of greenhouse-grown pepper was conducted by the authors of [29]. Hyperspectral data (400–1000 nm) were used to obtain the total correct classification in identifying early damage in the peppers.

Spectral studies of plants grown in aquaponic systems are very scarce. A team led by Mohamed Taha reported the nutrient deficiency determination and chlorophyll content in lettuce grown in an aquaponic system [30,31,32].

This study was conducted to address a gap in current research on the effects of varying exposure times to microelement fertilization in aquaponic systems, as there is limited understanding of how such treatments impact both plant growth and fish health while maintaining optimal water quality. Additionally, the use of VIS-NIR spectroscopy for the non-destructive assessment of plant nutrient status and stress in aquaponics has not been fully explored, making this study a valuable contribution to optimizing system management and crop monitoring in aquaponic practices.

This study aims to investigate the impact of varying exposure times to microelement fertilization on hydrochemical parameters, plant growth metrics, and microelement content in an aquaponic system cultivating pepper (*Capsicum annuum*) in integration with common carp (*Cyprinus carpio*); second, it aims to investigate the possibilities of visible–near-infrared spectroscopy to distinguish between different treated plants and pepper elements’ content.

## 2. Results and Discussion

### 2.1. Growth Parameters in Fish C. carpio

The data from Table 1 delineate how exposure to microelements, even at varying intervals, does not adversely affect the overall health and growth of common carp, as evidenced by a consistent 100% survival rate across all treatments. This finding is paramount as it suggests the non-toxic nature of the microelement treatment used and corroborates the notion that aquaponic systems can sustain healthy fish populations when microelements are administered within appropriate bounds.

The specific growth rate (SGR) observed at 2.67 ± 0.11%·day^−1^ signifies a positive growth performance and is an indicator that the fish were thriving. It suggests that the microelement treatment, across the exposure times tested, was within a range that supported optimal growth, echoing the conclusions reported by the authors of [18], who found that this specific growth rate is found in aquaponic systems without inducing stress.

Moreover, the Feed Conversion Ratio (FCR) of 1.56, while moderately higher than ideal, suggests that the microelement treatment did not diminish feed efficiency. The effects of different microelements on fish can significantly vary depending on factors such as dosage and exposure duration. An excellent example of this is the treatment of iron, which can lead to improved growth in fish species like swordtails (*Xiphophorus helleri*) and platyfish (*X. maculatus*). However, on the flip side, when iron is added continuously, it can become dangerous due to the risk of toxicity. This dual nature of iron’s impact is well documented in previous studies [33,34,35]. Microelements such as copper, iron, and zinc play an important role in fish growth. High levels of copper in water cause fish intoxication [36]. Cu can also cause changes in kidney cells, reduced growth, and a decrease in immune response [37]. Fe is required for fish growth and a high concentration can cause structural damage, subsequently affecting fish growth and survival [38]. According to Deswati et al. (2021), better concentrations of Cu, Fe, and Zn will accelerate fish and plant growth [39]. Atique and Pirhonen (2021) compared the survival indicators of fish grown in a recirculation system and aquaponics with added micronutrients (Fe, Cu, Mn, Zn, and B) to the aquaponic systems [40]. They established that the SGR of the fish was significantly higher in aquaponics (1.95 ± 0.12) than in RAS (1.67 ± 0.08). In addition, the FCR in aquaponics was significantly lower (0.85 ± 0.08) than in RAS (1.06 ± 0.03). However, the results also prompt further investigation into the specific microelement concentrations and their direct effects on fish metabolism and growth, as the nuances of these relationships are not yet fully understood.

The absence of negative impacts on fish growth and survival rates in our study is a promising indication that microelement treatments, if optimized, could enhance the productivity of aquaponic systems related to plants. This optimization, however, must consider the fine balance required in these systems, ensuring that the benefits seen in plant growth do not come at the expense of aquatic life.

### 2.2. Hydrochemical Parameters

The conductivity values observed in our study ranged from 271.66 ± 2.07 μS/cm to 297.66 ± 14.12 μS/cm across different treatments. This range indicates a relatively stable ionic concentration in the water, which is essential for the health of both plants and fish in aquaponic systems. As noted, optimal conductivity levels are crucial for nutrient absorption and overall system balance [41]. The slight increase in conductivity in the v-2 variant could be attributed to the accumulation of ions over time; specific interactions with microelements [42,43] determined that electrical conductivity significantly affects fish production. However, the return to baseline levels in the v-4 and v-6 treatments suggests a system self-regulation mechanism, which is probably connected with the influence of electrical conductivity on bacterial and archaeal communities.

The pH values in our study remained within a narrow range (7.28 ± 0.07 to 7.33 ± 0.05) (Table 2), indicating a stable and balanced aquatic environment. This stability is vital, as pH influences nutrient availability and fish health [44,45,46]. The minor fluctuations observed are within the acceptable range for aquaponic systems, supporting previous findings [47], which noted that minor pH variations are typical in such systems due to biological processes.

The levels of dissolved oxygen (DO) ranged from 6.17 ± 0.6 mg/L to 6.88 ± 0.56 mg/L (Table 2). The productivity in aquaponics is related to oxygen supply [48]. The slightly higher DO in the v-2 variant could be related to the treatment’s effects on microbial activity. However, all variants maintained DO levels that support healthy aquaponic system functioning.

The ammonia nitrogen levels showed some variation across treatments, with the lowest concentration observed in the v-2 treatment (0.144 ± 0.01 mg/L), which was significantly lower than the control (0.24 ± 0.02 mg/L) (Table 3). This reduction in ammonia levels is beneficial for the system, as high ammonia levels can be toxic to fish [49,50,51]. The levels observed in other treatments (v-4 and v-6) remained within a safe range for aquaponic systems; according to a previous study [52], the maximum acceptable concentration of ammonia and nitrite in common carp fry was estimated at 10.24 and 51.24 mg/L, respectively.

The nitrite levels varied slightly across treatments but remained within a relatively narrow range (0.18 ± 0.08 mg/L to 0.26 ± 0.12 mg/L). We did not find significant differences in its concentrations between different treatment variants. As nitrite is an intermediate product in the nitrification process, its stable levels across treatments suggest an efficient biofiltration process, which is crucial for fish health [53].

Nitrate levels decreased notably in treatments v-2, v-4, and v-6 compared to the control, with the lowest concentration in v-4 (4.18 ± 0.64 mg/L). As one of the main nitrogen forms used in plant nutrition [54], this reduction in different experimental treatments might be due to higher plant uptake. Nitrates are less harmful than ammonia or nitrite and are a key nutrient for plant growth, so their optimal levels, as discussed by Yang and Kim (2020), are crucial for balance in aquaponic systems [55].

There was a gradual decrease in orthophosphate levels across all treatments, with the lowest concentration in the v-6 treatment (2.31 ± 0.16 mg/L). The reduction in phosphate levels can be attributed to high Ca and Mg concentrations in this experimental variant, which might have caused the precipitation of phosphate salts [56]. Maintaining appropriate orthophosphate levels, as emphasized by Salim et al. (2019), is essential for plant growth [57].

### 2.3. Growth Parameters in Cultivated Plants

**Figure 1:**
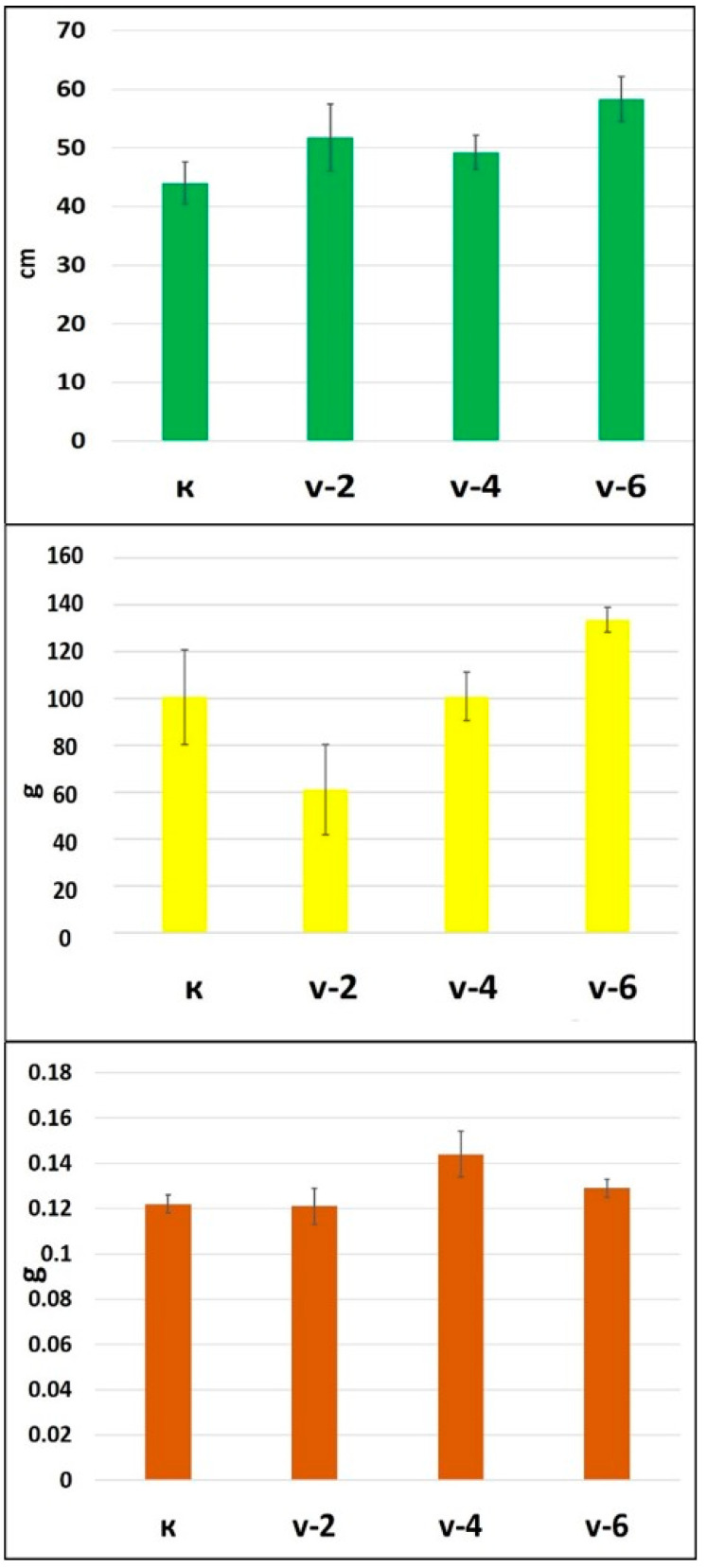
Average root length, weight, and yield of cultivated pepper.

Many reports on aquaponics have focused on aquaculture, but few studies have focused on the nutrient dynamics of plants [4]. In this study, the increase in the average root length in treatments 2 and 6 suggests (Figure 1) that certain microelement exposure times may be conducive to root development. The substantial root length in treatment 6 (58.33 cm) could indicate improved nutrient absorption capabilities, potentially translating to enhanced overall plant health and yield. The weight data present a more complex picture. The decrease observed in treatment 2 (61.2 g) contrasts with the root length data, suggesting that while the roots were longer, this did not correlate with an increase in biomass. This discrepancy might be explained by the partitioning of energy to root development over fruit mass. Conversely, treatment 6 not only showed the greatest root length but also the highest weight (133.4 g), indicating a positive relationship between root development and fruit biomass when exposed to the optimal duration of microelement treatment.

Yield data further complicate the picture. Treatment 2, despite its lower weight, did not show a significant drop in yield (0.121 g), which was nearly equivalent to the control (0.122 g). This could suggest that the number of fruits produced was not affected, or that the size of individual fruits was smaller, impacting total weight but not yield. Treatment 4 presented the highest yield (0.144 g), indicating that there may be a peak exposure time for maximizing yield before any potential phytotoxicity or nutrient imbalances affect the plant.

Taken together, these data points suggest that there is a complex interplay between microelement exposure time and plant growth parameters in aquaponic systems. The optimal microelement exposure time that promotes root development does not necessarily correspond to increased plant weight or yield, as evidenced by the data from treatment 2. However, treatment 6 appears to strike a balance, supporting both robust root growth and increased plant weight, which is indicative of healthy plant development and a potentially greater nutritional value.

These observations underscore the importance of fine-tuning microelement exposure to achieve desired growth outcomes in aquaponic systems. While increased root length is generally positive, the ultimate goal is to maximize both the biomass and the yield of the plants, which treatment 6 achieved most effectively. These insights are valuable for the design of future aquaponic systems and the development of nutrient management protocols that optimize plant growth and productivity.

### 2.4. Macro- and Microelements

In Table 4, the results of the multivariate analysis of macro- and microelement content were presented, depending on the different treatment variants for seedlings. Significant differences (*p* < 0.05) were observed in the manganese content between the control and all other treatment variants. Notably, significant differences were calculated for the microelements borum and ferrum between the control and variant v-4, while zinc content showed differences between the control variant and variants v-2 and v-6.

Regarding the content of macroelements, the results show that nitrogen levels are highest in variant 4 (v.4). Figure 1 presents the yield parameters, with the highest results being observed in this variant. Nitrogen stimulates vegetative growth. The increase in yield in v.4 is likely due to the accumulation of higher contents of essential elements such as potassium and boron, which contribute to the formation of cell walls in the plant.

Calcium plays a role in the development of the root system and the biomass growth of peppers, and in this study, the highest values for the parameters of stem length and biomass were recorded in variant 6 (v.6). Calcium is a key element for plant growth, as also noted in a previous study [58]. Manganese is a crucial micronutrient for plant nutrition, playing a role in photosynthesis. Significant differences in manganese levels were found between the control variant and the exposed variants. In v.6, the manganese content increased by 279.23% compared to the control [59], and a quadratic regression was observed between high-quality fruit yield and Mn concentration in peppers. Fe is involved in forming chlorophyll, which is necessary for light absorption during photosynthesis [60]. Manganese is involved in the processes of photosynthesis and protein synthesis [61]. These elements do not move easily from the leaves to other plant parts. If the cultivation medium lacks trace elements, deficiency symptoms are observed in the photosynthetically active leaves and plant growth is suppressed [61]. In the case of zinc deficiency in the medium, plant growth is suppressed due to the relationship between zinc and the plant hormone auxin [62]. Phosphorus is an important element as it is involved in energy generation and enzyme activation, which are components of the phosphate compounds of biomembranes and DNA [63]. According to Kaburagi et al. 2024, a sufficient yield of Swiss chard could be obtained by adding 50% of the microelement level of the control solution when utilizing fish wastewater for aquaponics [64].

### 2.5. Spectral Characteristics of Tested Plants and SIMCA Classifications

**Figure 2:**
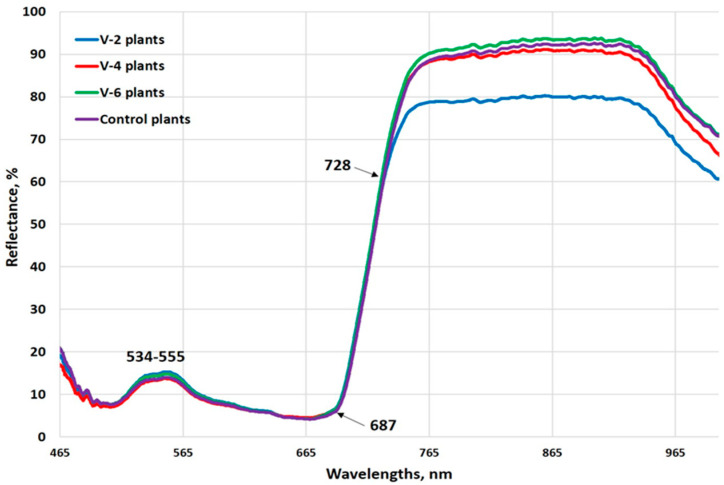
Average reflectance spectra of pepper leaves in the visible–short wave NIR region.

The spectral characteristics of the investigated pepper leaves can be seen in Figure 2. In the visible region, the spectra of control and treated plants were very similar, with the biggest absorption in the blue and red regions, due to chlorophyll. The lowest reflectance (the highest absorbance) was observed around 670 nm. Small differences existed in the green region (534–555 nm). The spectral reflectance rose sharply at the “red edge” (680–750 nm). In the near-infrared range, the reflection of v-2 variant plants was lower than that of the other tested plants. The reflectance of the v-4 and v-6 variants, as well as the control plants, was very similar.

SIMCA models for the discrimination of control and treated plants were developed. The best models were obtained using smoothing and the first-derivative transformation of spectral data. The results of the developed SIMCA models are presented in Table 5. The total misclassification rate was 1.6%. The F1-score combines precision and sensitivity into a single measure. The F1-score for different classes varied from 96.15 to 99.09%. The results showed the high performance of the developed SIMCA model for classification. Taha et al. (2022) reported a similar classification accuracy in the investigation of the estimation of full nutrition, nitrogen deficiency, phosphorous deficiency, and potassium deficiency of lettuce grown in aquaponics based on RGB images. Using different classification methods, they obtained an accuracy ranging from 79.8 to 96.5% [30].

**Figure 3:**
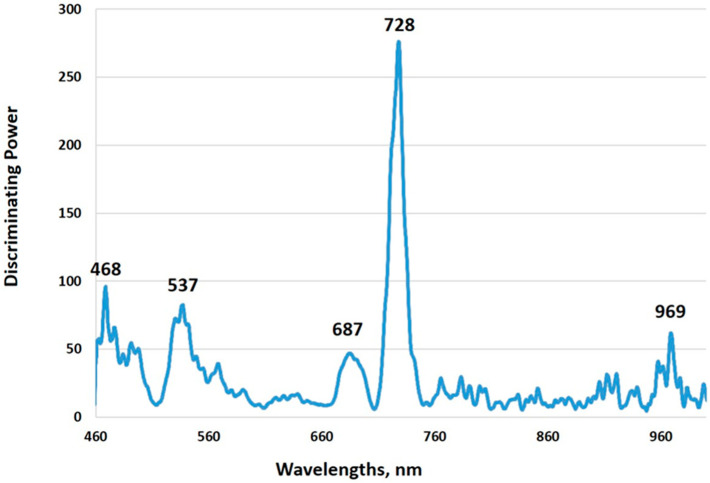
Discriminating power plot of SIMCA models for the discrimination of control of differently treated pepper plants.

The discrimination power plot shows the contribution of spectral information at different wavelengths to discriminating among classes. According to the discrimination power plot (Figure 3), the most important wavelengths for distinguishing classes of the studied pepper plants are 468 nm, 537 nm, 687 nm, 728 nm, and 969 nm. The most important wavelength for discriminating classes was 728 nm, corresponding to the red edge region of pepper leaves spectra. The wavelength of 687 nm marked the beginning of a sharp reflectance rise in the spectra. The 537 nm and 969 nm wavelengths are close to the positions where differences were observed in the spectra of the investigated plants. Similar wavelength regions were reported by other authors in the investigation of the determination of nitrogen, chlorophyll, and other pigments in plants. For example, Steidle Neto et al. 2017 reported the importance of the determination of chlorophyll, carotenoid, and anthocyanin in lettuce wavebands at 550, 690, and 950–1000 nm. Similar spectral information at 550 and 700–750 nm was used for the determination of chlorophyll in green tea leaves [65]. Kaburagi et al. (2020) and Steidle Neto et al. (2017) also reported 720–730 nm wavebands for the determination of chlorophyll in maize leaves [65,66].

The tested micronutrient fertilizer contained elements connected with photosynthetic pigments and the promotion of plant growth. For example, manganese aids the photosynthesis process; zinc and copper are a component of enzymes; boron is connected with the generation of cell walls; iron regulates and promotes growth; and molybdenum assists the formation of protein. The analysis of the chemical element content in the tested pepper leaves showed statistically significant differences in manganese, boron, iron, and zinc among the control and variants of the treated plants. These differences influence plants’ physiological conditions and pigment content, which, on the other hand, affects the spectral characteristics of the tested pepper plants. The spectral information allowed for a very good discrimination among differently treated plants [59,67].

Table 6 shows the statistics for the NIRS equations for the prediction of the mineral content of pepper leaves. Calibration and cross-validated correlation coefficients varied from 0.795 to 0.958, while RPD values varied between 1.91 and 3.50. An RPD value of 2–2.5 indicates that the resulting model is good enough, while a value of 2.5 or more than 3 implies a very good accuracy of determination. According to these criteria, the prediction ability of N, K, Ca, Mo, and S content based on the spectral characteristics of the leaves was very good. A good accuracy of determination was obtained for Mg, Mn, Cu, Fe, and Zn content. Only the accuracy of P determination was low.

The results for the determination of N and K content are consistent with those reported by Taha [30]. The difference in accuracy of P determination can be explained by the different spectral range used in the study.

In summary, the near-infrared spectroscopy methods presented in this study can be used to periodically non-destructively measure the nutrient concentration of pepper grown in aquaponics systems or other leafy plant cultivars to maintain the balance and sustainability of the system.

## 3. Materials and Methods

### 3.1. Experimental Aquaponic System

**Figure 4:**
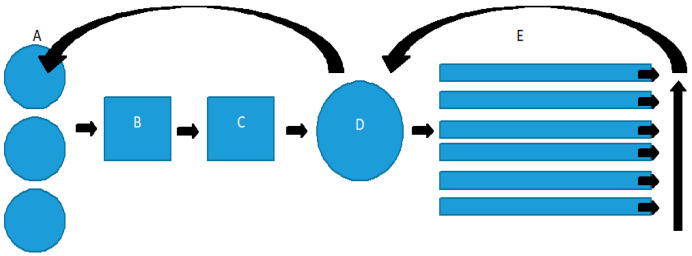
Schematic of experimental aquaponic system. (A) Fish tanks, (B) mechanical filter, (C) biofilter, (D) sump, and (E) plant section. Black arrows show the path of water.

For the experiment, we utilized an innovative aquaponic system located within a greenhouse spanning an area of 30 m^2^. This system comprised fish tanks, a mechanical filter/sedimentation tank (1 m^3^), a moving bed biofilter (1 m^3^), a sump, and eight plant raft tanks. Favorable climatic conditions in the greenhouse were maintained by a climate control system (Fujitsu AOYG30LFT). Oxygen was continuously supplied to both the fish and plant sections by two aerators, each with a capacity of 110 L/min (Hailea group, China). A schematic of the system used can be seen in Figure 4.

### 3.2. Experimental Fish and Plants

Healthy common carps, without visible injuries, were transported from the Tundja 73 farm in Nikolaevo, Bulgaria. They were weighed and stocked in the aquaponic system at a density of 22 kg·m^−3^. The experiment started after a 10-day acclimatization period.

Throughout the trial, fish mortality was monitored daily. To conclude, the survival rate (%) of the fish was calculated with the following formula:

Survival rate (%) = Nt/No × 100

Additionally, at the end of the trial, the specific growth rate (SGR) and Feed Conversion Ratio (FCR) were determined using the following equations:

SGR%.d − 1 = log〖Final weight − log〖initial weight〗〗/(Experimntal periods in days) × 100

Feed conversion ratio (FCR) = (Feed fed)/Gain(1)

**Figure 5:**
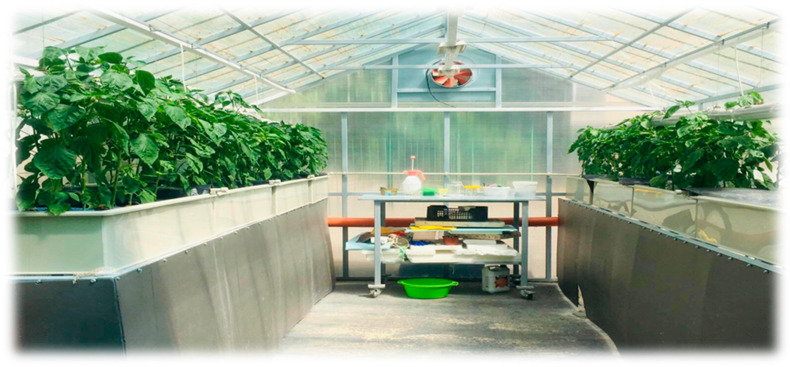
Image of experimental greenhouse used in current trial.

Seedlings of the pepper variety ’Sladka Shipka’ were transported from a greenhouse in the Plovdiv area to the aquaponic system at the Faculty of Agriculture, Trakia University, Stara Zagora. The seedlings were transplanted in hydroponic pots filled with Lightweight Expanded Clay Aggregate (LECA) and were placed in the plant growing beds in the plant section (Figure 5).

Seedlings were fertilized with liquid microelements twice weekly. We utilized the “Oligo Spectrum” (DTPA iron, EDTA manganese, EDTA zinc, sodium molybdate, citric acid, potassium carbonate, and cobalt sulfate) for microelement fertilization with exposure times of 2, 4, and 6 h. The concentration used was 5 mL·L^−1^, as recommended by the manufacturer. The experimental variants were labeled as follows: v-2, v-4, and v-6. During the treatment, the water flow to the plant growing beds was halted. After the treatment period ended, the water in them was drained, and the water supply was restored. There was also a control variant (k) that had no exposure time to the preparation. The quantity of added fertilizer was determined according to the producer’s requirement—2 mL·L^−1^. The experiment was conducted in duplicate, and each experimental variant consisted of 12 pepper plants. The plants were introduced at the 7th–8th true leaf stage and the experiment was interrupted when the 2nd–3rd fruit phase demonstrated typical size and shape.

At the end of the experiment, we measured the average root length (cm) to the nearest millimeter using a ruler, as well as the average weight of the plants (g) and the average yield (g) from each experimental variant using a technical balance BIMCO B3 F. The accuracy of these measurements is 0.1 g.

### 3.3. Determination of Hydrochemical Parameters

Conductivity, pH, and dissolved oxygen were measured using the portable lab meter HQ-30D (Hach, Loveland, CO, USA) every day. Other hydrochemical parameters—ammonia nitrogen (Method 8038 Nessler Method), nitrite (Method 10,019 Diazotization Method Test ‘N Tube™ Vials), nitrate (Method 8039 Cadmium Reduction Method Powder Pillows or AccuVac^®^ Ampuls), and orthophosphate (Method 8178 Amino Acid Method)—were measured weekly using a DR2800 spectrophotometer (Hach) according to the manufacturer’s instructions [68].

### 3.4. Determination of Macro- and Microelements

For the purpose of macro- and microelement analysis, 3 × 200 g foliar samples from each tested experimental variant was assured and transported after preparation to an accredited laboratory. The macro- and microelement content of the pepper was analyzed at independent accredited laboratories—Yara Analytical Services and Lancrop Laboratories (York, UK)—using the following methods:

➢ Total N—based on Dumas method N11 using LECO CNS analyzer, as described by Culmo (2010) [69].
➢ Boron, calcium, copper, iron, potassium, magnesium, manganese, molybdenum, phosphorus, sulfur, zinc—method 1.17 using microwave digestion with nitric acid; analysis by ICP-OES, as described by Kane et al. (2006) [70].

### 3.5. Spectral Measurements

The spectral measurements were taken in the wavelength interval of 450–1100 nm using a USB4000 (Ocean Optics, Inc., Dunedin, FL, USA) spectrometer and a fiberoptics probe for reflectance measurements. The probe consists of seven optical fibers—six of them lead the light from a stabilized halogen light source to the measured surface and one of them leads the reflected light to the spectrometer.

In order to avoid wilting, the plants were kept in pots filled with water from the aquaponic tanks. The measurements were performed without tearing the leaves from the plants. Several leaves from each plant were chosen for spectral measurements. The distribution of the leaves along the stem was as follows: three leaves nearest to the root, three leaves around the first branch of the stem, and three leaves at the top of the plant. Every leaf was measured in three different circular areas, each of them with a diameter of 6.5 mm. The spectra of each plant were then averaged. The measurements were performed in an air-conditioned room at a temperature between 25 °C and 28 °C.

### 3.6. Multivariate Data Analysis

Differences in the macro- and micronutrient content of seedlings based on the treatment variants were calculated with multivariate ANOVA at *p* < 0.5. Data analysis was carried out using the statistical software IBM SPSS Statistics 26.0 (New York, NY, USA).

Pirouette 4.5 software (Infometrix, Inc., Bothell, WA, USA) was used for the spectral data processing. Soft Independent Modeling of Class Analogy (SIMCA) was applied to develop the classification models. In SIMCA, each different class was described by its own model, and was based on principal components analysis (PCA). Samples were divided into four classes—control, along with experimental variants v-2, v-4, and v-6. Different mathematical treatments of spectral data were evaluated—smoothing, multiple scatter correction, and first and second derivatives. The evaluation of developed models was based on statistical parameters—precision, sensitivity, F1-score, and misclassification rate. Precision measures the ability of the model to identify samples from a particular class. It is calculated as the number of correct predicted samples from the respective class divided by the total number of samples of that class. Recall is the number of correct predicted samples from a particular class divided by the sum of correct predicted samples plus samples from other classes, predicted as belonging to that class. The F1-score is a harmonic mean of precision and sensitivity (recall) calculated for each class. In a multi-class classification model, the F1-score for the class is a digital representation of whether the prediction on a specific class is valid. These indicators were multiplied by 100 to become a percentage. PLS regression was used to develop equations for plants’ mineral content determination. The calibration equations for each parameter were developed and validated with leave-one-out cross-validation. The prediction capacity of each calibration equation was evaluated using statistical parameters from the calibration procedure, e.g., R—multiple correlation coefficients between reference values and NIR predicted values; SEC—standard error of calibration; SECV—standard errors of cross-validation; and RPD—the ratio between the standard deviation of a particular element and SECV.

## 4. Conclusions

No clear trend was found in the influence of exposure time to Oligo Spectrum^®^ on the cleaning capacity of raft tanks in the experimental aquaponic system. Experimental variant 2 showed the strongest cleaning capacity for ammonia nitrogen. Nitrates were best managed in experimental variant 4, and orthophosphates by the variant with a 6 h exposure time of microelements in plant tanks.

There was no detected negative impact of treatment with the preparation on the survival and growth of common carp (*C. carpio* L.) in the aquaponic system. The average weight of cultivated plants peaked in the variant with a 4 h exposure time of micronutrients in the plant section tanks. The longest root length and highest average yield were observed in experimental variant 6.

The difference in the chemical composition and physiological state of pepper plants affects their spectral characteristics in the visible and near-infrared regions in a specific way. This information can be used to develop models for the non-destructive determination of the nutritional status of plants.

## Figures and Tables

**Table 1:** Growth parameters in *C. carpio*.

Parameters	
Average initial weight (kg)	0.256
Average final weight (kg)	0.388
Survival (%)	100
SGR (%)	2.67 ± 0.11
FCR	1.56

**Table 2:** Conductivity (µS/cm), pH, and dissolved oxygen (mg·L^−1^) in plants during the trial.

	Control	v-2	v-4	v-6
Conductivity	271.66 ± 2.07	297.66 ± 14.12	272 ± 1.61	271.66 ± 1.47
pH	7.33 ± 0.05	7.28 ± 0.07	7.31 ± 0.03	7.31 ± 0.07
Dissolved oxygen	6.17 ± 0.6	6.88 ± 0.56	6.56 ± 0.51	6.58 ± 0.70

**Table 3:** Nitrogen and phosphate compounds (mg·L^−1^) during the trial.

	Control	v-2	v-4	v-6
Ammonium nitrogen (NH_4_^+^)	0.24 ± 0.02	0.144 ± 0.01 *	0.18 ± 0.01	0.26 ± 0.008
Nitrite (NO_2_^−^)	0.18 ± 0.8	0.26 ± 0.12	0.22 ± 0.14	0.26 ± 0.12
Nitrate (NO_3_^−^)	8.06 ± 3.31	5.56 ± 1.90	4.18 ± 0.64 **	4.52 ± 0.99 *
Orthophosphate (HPO_4_^2−^)	2.80 ± 0.43	2.48 ± 0.27	2.45 ± 0.21	2.31 ± 0.16 *

* *p* ≤ 0.05; ** *p* ≤ 0.01.

**Table 4:** Multivariate ANOVA of macro- and micronutrient content based on the different variants of treatment.

	к	v-2	v-4	v-6
Nitrogen (N)	4.575 ± 0.08	4.120 ± 1.40	4.237 ± 1.05	4.175 ± 1.14
Phosphorus (P)	0.280 ± 0.04	0.225 ± 0.12	0.235 ± 0.09	0.225 ± 0.09
Potassium (K)	2.555 ± 0.43	4.045 ± 0.49	4.253 ± 0.77	3.533 ± 0.71
Calcium (Ca)	4.355 ± 0.22	3.840 ± 1.64	4.048 ± 1.17	4.028 ± 0.99
Magnesium (Mg)	0.640 ± 0.01	0.760 ± 0.325	0.780 ± 0.25	0.70 ± 0.24
Manganese (Mn)	131.45 ± 5.87 ^a^	326.10 ± 73.96 ^a^	322.10 ± 46.89 ^a^	367.05 ± 80.30 ^a^
Borum (B)	36.70 ± 4.38 ^a^	41.85 ± 0.49	49.85 ± 4.52 ^a^	46.475 ± 3.32
Cuprum (Cu)	5.55 ± 0.78	4.80 ± 1.70	5.176 ± 1.36	4.575 ± 0.99
Molybdenum (Mo)	4.035 ± 0.58	5.02 ± 2.99	5.158 ± 2.12	6.055 ± 2.74
Ferrum (Fe)	68.0 ± 4.24 ^a^	78.0 ± 9.90	81.75 ± 1.50 ^a^	75.25 ± 7.36
Sulfur (S)	0.435 ± 0.04	0.37 ± 0.13	0.388 ± 0.09	0.383 ± 0.11
Zinc (Zn)	100.8 ± 2.83 ^a^	78.45 ± 10.39 ^a^	90.85 ± 12.35	78.95 ± 9.43 ^a^

^a^ the same superscript within the same row represents significant differences at *p* < 0.05.

**Table 5:** Results of SIMCA models for the discrimination of control of differently treated pepper plants.

	v-2	v-4	v-6	Control	Precision, %	F1-Score, %
v-2	50	4	0	0	92.6	96.15
v-4	0	108	0	0	100	98.18
v-6	0	0	109	0	100	99.09
Control	0	0	2	108	98.2	99.08
Sensitivity, % (Recall)	100	96.43	98.20	100		

**Table 6:** Calibration statistics for the NIRS estimation of the mineral content of pepper leaves using PLS regression.

Parameter	SECV	Rcv	SEC	Rcal	RPD
N, %	0.296	0.917	0.291	0.921	3.13
P, %	2.236	0.795	2.180	0.809	1.91
K, %	0.256	0.957	0.254	0.958	3.31
Ca, %	0.398	0.935	0.378	0.942	3.11
Mg, %	0.078	0.925	0.077	0.928	2.71
Mn, ppm	38.50	0.944	37.47	0.944	2.59
B, ppm	2.269	0.921	2.260	0.922	2.57
Cu, ppm	0.383	0.911	0.375	0.916	2.89
Mo, ppm	0.611	0.914	0.601	0.918	3.50
Fe, ppm	2.671	0.920	2.660	0.922	2.65
S, %	0.027	0.022	0.026	0.925	3.30
Zn, ppm	4.290	0.932	4.215	0.936	2.88

## Data Availability

Data is contained within the article.
